# Active Site Loop Dynamics of PriB, a C-prenyltransferase

**DOI:** 10.1063/4.0001118

**Published:** 2025-10-27

**Authors:** Omowumi Oreoluwa Fagbohun, Jonathan Clinger

**Affiliations:** Baylor University, Waco, Texas, USA

## Abstract

Numerous biological functions are demonstrated by prenylated natural compounds, such as prenylated indole analogs. Natural product biosynthesis and modification of structures are significantly influenced by prenyltransferases. The transfer of prenyl groups from prenyl donors to indole-containing compounds is catalyzed by the enzyme C-prenyltransferase (PriB). PriB has been identified as carbon-6 C-prenyltransferase of indoles, utilizing pyrophosphates as prenyl donors. Prenyl group attachment confers benefits to a variety of naturally occurring compounds, such as increased affinity for binding of target proteins and increased interaction with cell membranes. In the structures of PriB, there’s a loop that covers the active site, which closes upon ligand binding and opens in the absence of ligand (apoPriB). X-ray crystallography was used to study the loop dynamics of PriB, and protein engineering was performed to know the importance of a loop residue in the enzyme’s catalysis. PriB was co- crystallized and soaked with tryptophan, generating one tryptophan and two tryptophan-bound structures, respectively. Single x-ray crystallography of PriB in its apo form revealed the absence of electron density in the loop covering the active site. Datasets of PriB with one tryptophan and two tryptophans were also collected, with the main chain of the second tryptophan of the two tryptophans dataset partially occupying the position of the actual second substrate, dimethylallylpyrophosphate (DMAPP). The structure of PriB with one tryptophan showed the electron density of this loop covering the active site, but this loop appears to be disordered compared to the structure of PriB with two tryptophans. This sheds light on the flexibility of this loop with or without substrate bound. Also, the position of this loop in the structure of PriB with tryptophan appears to be open compared to the previously deposited PDB structure of PriB with tryptophan and dimethylallyl S- thiolodiphosphate, a mimic of DMAPP. Future studies on this enzyme will give deep insight into the structural transitions of PriB.

